# Retraction Note: MiR-106b and miR-93 regulate cell progression by suppression of PTEN via PI3K/Akt pathway in breast cancer

**DOI:** 10.1038/s41419-026-08631-2

**Published:** 2026-03-20

**Authors:** Nana Li, Yuan Miao, Yujia Shan, Bing Liu, Yang Li, Lifen Zhao, Li Jia

**Affiliations:** College of Laboratory Medicine, Dalian Medical University, Dalian, Liaoning Province China

Retraction Note to: *Cell Death & Disease* 10.1038/cddis.2017.119, published online 18 May 2017

The Editors-in-Chief have retracted this article. Concerns were raised regarding a number of figures, specifically:

- Figure 2j: the panels for anti-miR-106b and anti-miR-93 appear to partially overlap.
- Figure 6a: panel 3 (left) and panel 3 (right) for MDA-MB-231 appear to partially overlap.

The Editors-in-Chief therefore no longer have confidence in the reliability of the data reported in the article.

Author Li Jia has stated on behalf of all authors that they disagree with this retraction.

